# Rational Design and Characterization of a Mutated Nanobody for Specific Targeting of Heparan Sulfate

**DOI:** 10.3390/antib15040052

**Published:** 2026-06-23

**Authors:** Junfang Hao, Qian Xu, Yanyan Cui, Wenlong Wang, Kai Huang

**Affiliations:** 1 College of Biology and Food, College of Smart Animal Husbandry, Shangqiu Normal University, Shangqiu 476000, China; cuiyanyan@sqnu.edu.cn (Y.C.); wangwenlong202604@163.com (W.W.); 2 Department of Preventive Veterinary Medicine, College of Veterinary Medicine, Northwest A&F University, Yangling 712100, China; 15290032037@163.com; 3 Zhoukou Municipal Administration for Market Regulation, Zhoukou 466000, China; huangkai1739@163.com

**Keywords:** nanobody, heparan sulfate, rational design, virus attachment

## Abstract

Background: Viral attachment mediated by host cell surface receptors is the first step in viral infection. As a key cell surface receptor, heparan sulfate (HS) mediates the attachment and entry of numerous non-enveloped viruses in livestock, thereby serving as a crucial molecular target for studying virus–host interactions. Methods: Based on the structural scaffold of a nanobody (Nb; PDB: 7TJC), we rationally designed and constructed a mutant Nb targeting HS, designated HS-Mut-Nb1, using molecular docking, site-directed mutagenesis, molecular dynamics (MD) simulations, and experimental characterization. Results: Molecular docking indicated that the active site of wild-type Nb for HS binding was located within the cavity jointly formed by the complementarity-determining region 3 (CDR3) and the framework regions (FRs) of the wild-type Nb. A comprehensive analysis integrating virtual alanine scanning, site-directed mutagenesis, and MD simulations revealed that the combination of three point mutations (Phe47Arg, Asp99Tyr, and Tyr108Pro) significantly enhanced the binding affinity of Mut-Nb1 for HS, with a calculated binding free energy (ΔG) of −83.26 ± 3.06 kcal/mol. Enzyme-linked immunosorbent assay (ELISA) results further confirmed that Mut-Nb1 exhibited high affinity for HS (*K*_D_ = 65.87 nM) and specificity (positive/negative ratio, P/N = 3.84; cross-reactivity, CR < 6.60%). Conclusions: This study not only provides novel candidate molecules for elucidating the mechanism of HS–virus interactions and developing related inhibitors but also offers a reference for the rapid construction of mutant Nbs.

## 1. Introduction

Viral infection is initiated by the binding of viruses to host cell surface receptors. Many non-enveloped viruses rely on interactions between their structural proteins and cell surface glycosaminoglycans (GAGs) to facilitate attachment [1]. Among these, heparan sulfate (HS) has attracted considerable attention due to its involvement in multiple critical stages of viral infection, including attachment, entry, and replication [2,3,4]. HS is a linear polysaccharide side chain of heparan sulfate proteoglycans (HSPGs) and is widely distributed on mammalian cell surfaces and within the extracellular matrix [5]. Under physiological conditions, its negative charge nature can effectively bind positively charged virions, thereby enhancing viral attachment efficiency and infectivity [6]. For instance, the capsid protein (Cap) of porcine circovirus type 2 (PCV2) directly interacts with HS to mediate viral attachment to the cell surface [7]. Studies using HS-deficient cells have shown that the absence of HS significantly inhibits dengue virus type 2 (DENV-2) attachment. In contrast, for Zika virus (ZIKV), HS is dispensable for attachment but plays a primary role in the post-attachment replication stage and contributes to virus-induced apoptosis [3]. Collectively, these findings reveal the pivotal role of HS in viral attachment and entry. Its structural diversity and widespread distribution make it a critical molecular target for understanding virus–host interactions and developing antiviral strategies.

Nanobodies (Nbs), also known as single-domain antibodies, are derived from the variable regions of camelid heavy-chain-only antibodies, with a molecular weight of approximately 15 kDa [8]. Owing to their small molecular size, ease of genetic manipulation, high specificity, and excellent solubility [9,10], Nbs have become essential molecular tools in veterinary science. They are employed in fundamental research, such as elucidation of protein–protein interaction mechanisms and in vivo tracing [11,12,13], and are widely used for the precise detection and diagnosis of pathogens [14,15,16]. Moreover, they can block microbial infection cycles or modulate host signaling pathways for therapeutic interventions [17,18,19]. However, research on Nbs targeting host cell surface molecules remains limited. Jähnichen et al. [20] developed Nbs specific for the chemokine receptor CXCR4. These Nbs not only exhibited potent antiretroviral activity against T-cell-tropic and dual-tropic HIV-1 strains but also promoted the migration of CD34^+^ stem cells. The nanobody Nb2, screened by Deng et al. [21] against the porcine reproductive and respiratory syndrome virus (PRRSV) receptor CD163, was shown to broadly inhibit diverse PRRSV lineages and downregulate the NF-κB pathway. Further research confirmed that its antiviral activity is achieved by blocking viral attachment and suppressing CD163 transcription. Notably, current Nb screening primarily relies on techniques such as phage display [22], ribosome display [23], and yeast display [24]. Nevertheless, these methods suffer from long screening cycles, high costs, and low activity of the resulting Nbs [25]. Protein structure-based computational virtual screening technology provides an innovative strategy for the rational design of mutant Nbs [26].

To the best of our knowledge, no molecular tools for the specific recognition of HS have been reported to date. In this study, by integrating molecular docking, site-directed mutagenesis, molecular dynamics (MD) simulations, recombinant protein expression and purification, and enzyme-linked immunosorbent assay (ELISA), we successfully generated a mutant Nb targeting HS (HS-Mut-Nb1). This work provides a novel candidate molecule for elucidating the mechanism of HS–virus interactions and developing related inhibitors, and it also offers a reference for the rapid design and screening of mutant Nbs.

## 2. Materials and Methods

### 2.1. Experimental Materials

HS, chloramphenicol (CAP), keratan sulfate (KS), and chondroitin sulfate (CS) were supplied by MedChemExpress (Princeton, NJ, USA), Sigma-Aldrich (St. Louis, MO, USA), Shanghai Aladdin Biochemical Technology Co., Ltd. (Shanghai, China), and Hebei Huayang Biotechnology Co., Ltd. (Hengshui, China), respectively. A mouse anti-His-tag monoclonal antibody and a horseradish peroxidase-conjugated goat anti-mouse IgG (goat anti-mouse IgG-HRP) were purchased from Proteintech Group, Inc. (Rosemont, IL, USA). Bovine serum albumin (BSA) and ovalbumin (OVA) were supplied by Sigma-Aldrich Trading Co., Ltd. (Shanghai, China).

Statistical analysis was performed using GraphPad Prism 10.4.2 (GraphPad Software, Boston, MA, USA).

### 2.2. Molecular Docking

First, the three-dimensional (3D) structural scaffold of the wild-type Nb was obtained from the RCSB Protein Data Bank (PDB, ID: 7TJC). For HS, the representative disaccharide repeating unit was selected as the minimal binding motif for docking, and its 3D structure was obtained from the PubChem database. Second, AutoDock 4 software was used to perform docking simulations between HS and the wild-type Nb. The grid box was centered at (X = 45.55, Y = 24.38, Z = 51.48), with dimensions of (X = 18 Å, Y = 18 Å, Z = 18 Å). Third, during the docking process, the maximum number of search conformers was set to 10,000. The genetic algorithm was employed for conformational sampling and scoring, and all other parameters were set to their default values. Finally, the conformations were ranked according to the docking scores, and the optimal conformation was selected.

### 2.3. Alanine Scanning and Site-Directed Mutagenesis

Based on the docking results, virtual alanine scanning was performed on the amino acid residues within a 5 Å radius of the HS binding site using Discovery Studio Visualizer 2019. Subsequently, site-directed mutagenesis was conducted on the key residues identified from the virtual alanine scanning analysis to validate their roles in HS binding.

### 2.4. MD Simulation

The point mutant model was constructed using PyMOL 2.5 (Schrödinger, New York, NY, USA) and served as the initial configuration for MD simulations. To ensure the reproducibility of the results, two independent replicate simulations were performed for each system, in which only the initial random velocities differed while all other parameters and conditions were kept identical. All simulations were performed using the GROMACS 2022.5 package. Before the simulation, the system was neutralized with NaCl counterions, and the complex was subjected to 2000 steps of energy minimization. Each system was subjected to a 100 ns MD simulation under periodic boundary conditions at 310 K and 1.0 bar. The minimum distance between the protein and the edge of the simulation box was set to 1.0 nm along the X, Y, and Z directions, with an integration time step of 2 fs. Energy minimization was performed using the steepest descent algorithm, and the cutoff distances for Coulomb interactions and van der Waals interactions were set to 1.4 nm. Based on the simulation trajectories, the root mean square deviation (RMSD), root mean square fluctuation (RMSF), radius of gyration (Rg), solvent-accessible surface area (SASA), and the number of intermolecular hydrogen bonds of the HS-Mut-Nb1 complex were calculated and analyzed. The binding free energy (ΔG) of the complex was calculated by the Molecular Mechanics Poisson–Boltzmann Surface Area (MMPBSA) approach for evaluating structural stability and intermolecular interaction intensity. Meanwhile, MMPBSA was applied to decompose the binding energy contribution of each key residue at the residue level.

### 2.5. Expression and Purification of the Recombinant Proteins

The gene encoding Mut-Nb1 was synthesized according to its amino acid sequence and cloned into the pET-28a expression vector. The recombinant plasmid was synthesized and constructed by General Biology (Anhui) Co., Ltd. (Chuzhou, China). The specific procedure for the induced expression and purification of the recombinant protein Mut-Nb1 was as follows: First, the recombinant Mut-Nb1 plasmid was transformed into *E. coli* BL21 (DE3) competent cells, and the transformed cells were inoculated into 5 mL of kanamycin-resistant LB liquid medium at a 1:100 dilution. Second, when the OD_600_ value reached 0.6–0.9, isopropyl β-D-1-thiogalactopyranoside (IPTG) was added to the medium to a final concentration of 1.0 mmol/L, followed by overnight induction at 15 °C with shaking at 200 r/min. Third, the cells were harvested by centrifugation at 10,000 rpm for 5 min at 4 °C, resuspended in PBS buffer (pH 7.4), and lysed on ice by sonication for 10 min until the suspension became clear. Then, the recombinant Mut-Nb1 was first enriched and purified via Ni-NTA affinity chromatography to remove most impurities. After concentration of the eluted fractions, further purification was performed by gel filtration chromatography (GFC). Finally, the purified Mut-Nb1 protein was assessed by sodium dodecyl sulfate-polyacrylamide gel electrophoresis (SDS-PAGE). Wild-type Nb was induced and expressed under the same conditions.

### 2.6. Affinity and Specificity Assays of Mut-Nb1

The binding affinity of Mut-Nb1 to HS was determined by ELISA. Briefly, the pre-conjugated HS-OVA and CAP-OVA were diluted with carbonate buffer solution (CBS, pH 9.6) to 2.0 μg/mL, coated onto ELISA plates at 100 μL/well (in triplicate), and incubated overnight at 4 °C. OVA served as a negative control. After coating, the plates were blocked with 1% (*w*/*v*) BSA at 37 °C for 1 h. Following this, the plates were incubated with purified Mut-Nb1 (10–0.02 μg/mL) at 37 °C for 30 min. An anti-His-tag monoclonal antibody (diluted 1:1000) was added, and the plates were incubated at 37 °C for 30 min. Then, goat anti-mouse IgG-HRP (diluted 1:1000) was added at 100 μL/well, and the plates were incubated at 37 °C for 30 min. Between each incubation step, the plates were washed five times with phosphate-buffered saline with Tween-20 (PBST) and blotted dry. For detection, 100 μL/well of 3,3′,5,5′-tetramethylbenzidine (TMB) substrate solution was added for color development. The reaction was stopped by the addition of 50 μL/well of 2 M H_2_SO_4_. The OD_450_ value of each well was immediately measured using a microplate reader. The equilibrium dissociation constant (*K*_D_) was determined by fitting the saturation binding curve with the one-site specific binding model in GraphPad Prism 10.4, using the nanobody concentration producing 50% of the plateau absorbance as the *K*_D_ value. Under this model, based on the monovalent nature of the nanobody-antigen interaction, the fitted half-maximal effective concentration (EC_50_) corresponds to the antibody concentration required to achieve half-maximal specific binding (Bmax/2) and is therefore reported as the intrinsic *K*_D_.

The specificity of Mut-Nb1 was evaluated by measuring its binding to structural analogs of HS. All analogs were coated at a fixed concentration of 2.0 μg/mL, and Mut-Nb1 was tested at a concentration of 2.5 μg/mL using the same ELISA method described above. Specificity was assessed using the following two parameters:

(1) Positive/negative (P/N) ratio: P/N = OD_450_ (positive control)/OD_450_ (negative control).

A P/N ratio ≥ 2.1 was considered specific binding; P/N < 2.1 was regarded as non-specific or negative.

(2) Cross-reactivity (CR%): CR% = [EC_50_ of HS]/[EC_50_ of CAP or HS structural analogs] × 100%.

The EC_50_ values of HS analogs were determined using the method described above. A lower CR% indicates weaker cross-recognition and thus higher specificity.

## 3. Results

### 3.1. Molecular Docking

**Figure 1:**
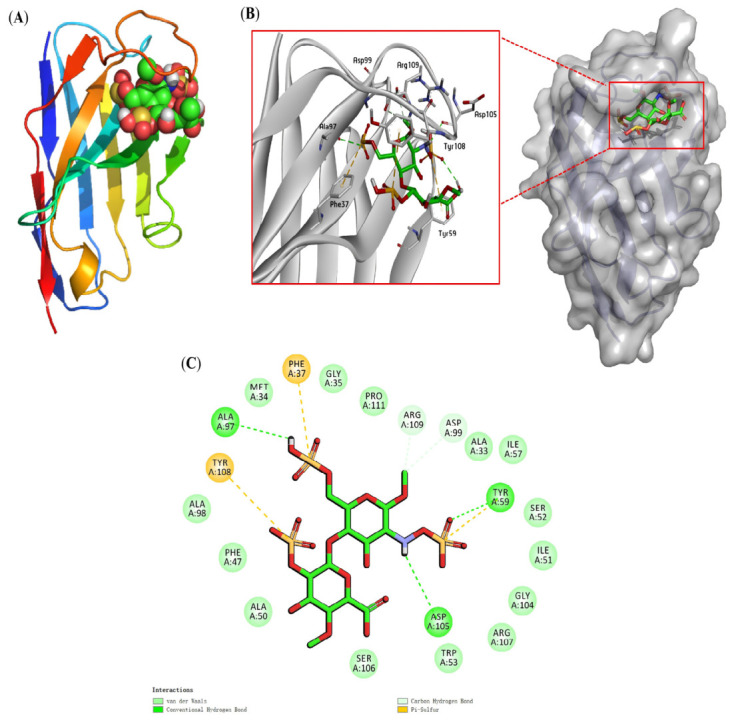
Molecular docking and interaction analysis of HS with wild-type Nb: (**A**) Molecular docking complex structure of HS and wild-type Nb (PDB: 7TJC). The Nb protein is represented as a cartoon model, while HS is depicted as a sphere model. (**B**) Interactions between HS and the active site in the cavity of wild-type Nb. The Nb is represented as a cartoon model, while HS is depicted as a ball-and-stick model. (**C**) Two-dimensional interaction diagram of HS with the active site of wild-type Nb. All figures were generated using Discovery Studio Visualizer 2019 and PyMOL.

Molecular docking results (Figure 1) showed that the active site for the binding of wild-type Nb to HS was located within the cavity formed by the complementarity-determining region 3 (CDR3) and framework regions (FRs) of wild-type Nb (Figure 1A), with a docking score of −7.30 kcal/mol (that of the Mut-Nb1 was −8.10 kcal/mol). This binding interaction was mainly mediated by key residues, including Phe37, Tyr59, Ala97, Asp99, Asp105, Tyr108, and Arg109 (Figure 1B). Specifically, the sulfonate groups of HS formed π–sulfur interactions with Phe37, Tyr59, and Tyr108 of wild-type Nb; stable conventional hydrogen bonds with Tyr59, Ala97, and Asp105; and carbon–hydrogen bonds with Asp99 and Arg109. Meanwhile, van der Waals forces were observed between HS and several residues surrounding the cavity (Figure 1C). These results indicated that the binding interactions between HS and wild-type Nb were predominantly driven by hydrogen bonds, π–sulfur interactions, and van der Waals forces.

### 3.2. Virtual Alanine Scanning

The virtual alanine scanning results indicated that the five residues, Phe47, Tyr59, Arg109, Phe37, and Tyr108, exhibited relatively high mutation energies (>0.5 kcal/mol) with a predicted destabilizing effect, suggesting that these residues are key contributors to the interaction between HS and wild-type Nb (Table 1). Notably, although Asp99 had a relatively low mutation energy (<0.5 kcal/mol), the absolute value of its electrostatic term (−0.33) was considerably higher than that of other residues, indicating that this residue plays a critical role in the electrostatic stabilization of the binding interface between HS and wild-type Nb. Therefore, these six residues were identified as critical for the interaction between HS and wild-type Nb and were selected for subsequent site-directed mutagenesis.

### 3.3. Site-Directed Mutagenesis

The results of single-site and double-site saturation mutagenesis are presented in Table 2 and Table 3, respectively. A total of 6 × 20 = 120 mutant variants were designed for single-site saturation mutagenesis, whereas C_6_^2^ × 20 × 20 = 6000 combinatorial variants were constructed for double-site saturation mutagenesis. Data from single-site saturation mutagenesis demonstrated that mutations of Asp99 to Phe, Gln, Asn, Tyr, Leu, or Cys; Tyr108 to Phe, Pro, or Val; and Phe47 to Arg enhanced the binding affinity between HS and wild-type Nb.

Based on the results of single-site saturation mutagenesis, triple-site combinatorial mutagenesis was performed, yielding a total of 1 × 7 × 3 = 21 mutant variants (Table 4). Analysis of the results from both double-site saturation mutagenesis and triple-site combinatorial mutagenesis revealed that the triple mutant Phe47 → Arg, Asp99 → Tyr, and Tyr108 → Pro constitutes the optimal variant for promoting the binding affinity between HS and wild-type Nb.

### 3.4. MD Simulations

**Figure 2:**
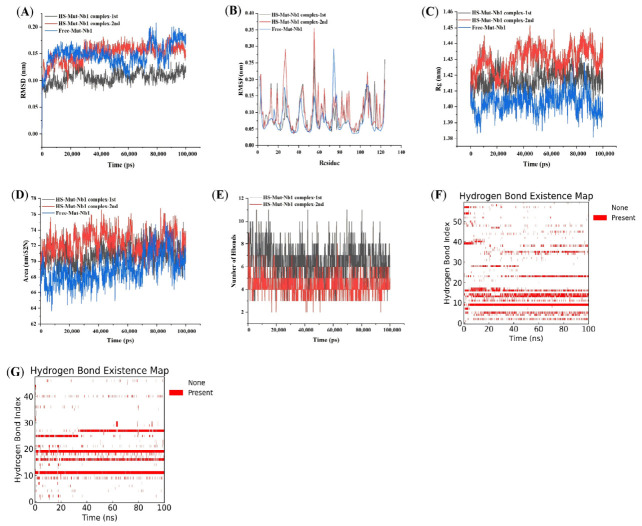
MD simulation results of the HS-Mut-Nb1 complex: (**A**) Root-mean-square deviation (RMSD). (**B**) Root-mean-square fluctuation (RMSF). (**C**) Radius of gyration (Rg). (**D**) Solvent-accessible surface area (SASA). (**E**) Number of hydrogen bonds. (**F**,**G**) Hydrogen bond existence maps ((**F**) 1st simulation; (**G**) 2nd simulation). Simulations were performed using GROMACS 2022.4 with the CHARMM36 force field.

In the 100 ns MD simulations, the conformational stability of the HS-Mut-Nb1 complex was assessed by RMSD (Figure 2A). The RMSD values of both independent simulations decreased rapidly during the initial stage and then reached a plateau. The second simulation (2nd) showed a slightly higher overall RMSD level than the first (1st), but both exhibited stable fluctuations, indicating that the complex had reached dynamic equilibrium. In comparison, the overall RMSD fluctuation range of the free Mut-Nb1 was 0.15–0.20 nm, which was markedly larger than that of the complexes in the two simulation groups. This result demonstrated that HS binding effectively restricted the flexible motion of the Mut-Nb1 backbone and improved the conformational stability of the complex system. RMSF analysis revealed that the residue flexibility profiles of the two simulations were highly consistent, with only minor differences of approximately 1 Å at individual residues, demonstrating the high reproducibility of the conformational flexibility of the HS-Mut-Nb1 complex (Figure 2B). Further analysis showed that residue His55 of Mut-Nb1 exhibited the most pronounced fluctuation, which is consistent with theoretical expectations. This can be attributed to the location of His55 within the CDR loop region, which inherently possesses high flexibility. By contrast, the free Mut-Nb1 exhibited a lower overall RMSF baseline, indicating it adopted a relatively compact conformation in the apo state. Rg and SASA results showed that the free Mut-Nb1 exhibited lower values, whereas the two simulations of the complex demonstrated highly consistent molecular compactness and surface exposure. The differences between the two simulations were approximately 0.1 Å for Rg and approximately 2 Å for SASA, both within the range of statistical error, further validating the structural stability of the HS-Mut-Nb1 complex (Figure 2C,D). The total number of hydrogen bonds fluctuated over time, with an average of 5–6 hydrogen bonds (Figure 2E). Hydrogen bond existence maps revealed 2–3 core stable hydrogen bonds that persisted throughout the entire simulation, indicating that the HS small molecule remained stably bound within the Mut-Nb1 protein cavity and formed stable interactions with key amino acid residues (Figure 2F,G).

**Figure 3:**
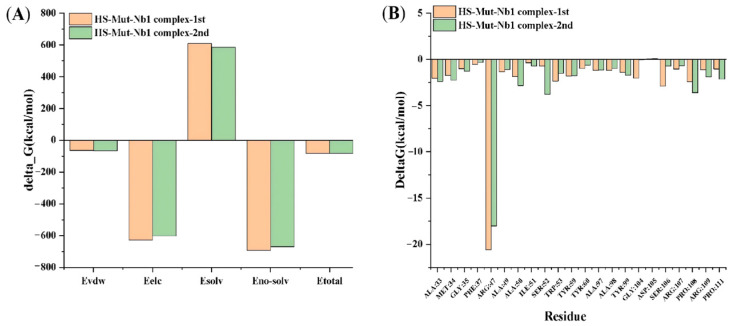
Binding free energy analysis of the HS-Mut-Nb1 complex: (**A**) Decomposition of binding free energy. (**B**) Key residues contributing to binding energy.

The binding free energy decomposition results from two independent simulations (Figure 3A) showed that the contribution trends of individual energy components were highly consistent, with total binding free energies (ΔG) of −83.14 ± 3.86 kcal/mol and −83.37 ± 1.95 kcal/mol (average: −83.26 ± 3.06 kcal/mol). This indicated that the HS-Mut-Nb1 complex exhibited strong thermodynamic binding affinity, further confirming the stability of the HS-Mut-Nb1 interaction from an energetic perspective. Analysis of key energy-contributing residues (Figure 3B) demonstrated that the Arg47 mutation provided the dominant energetic contribution in both simulations (ΔΔG = −20.59 and −18.01 kcal/mol, respectively), while the contributions of other key residues were relatively minor.

### 3.5. Expression and Purification of the Recombinant Protein

**Figure 4:**
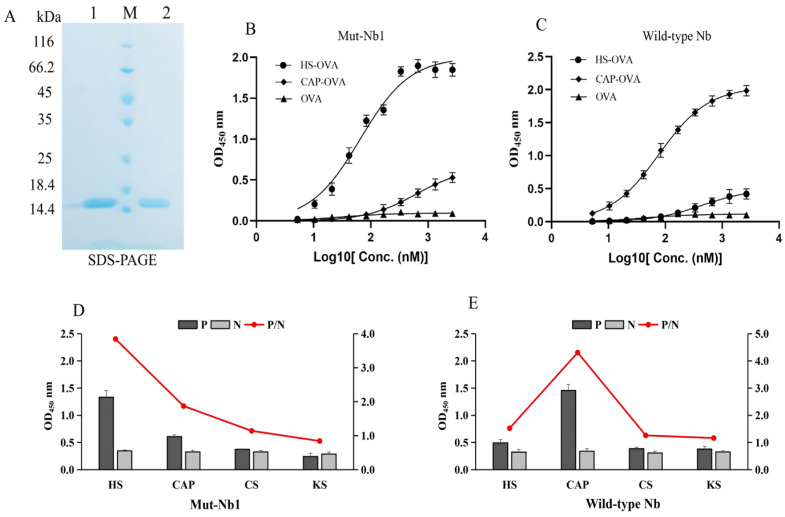
Characterization of purification, affinity, and specificity for recombinant proteins: (**A**) Purification of the recombinant Mut-Nb1 and wild-type Nb proteins. M: protein marker; Lane 1: recombinant Mut-Nb1 protein; Lane 2: recombinant wild-type Nb protein. (**B**–**E**) Affinity and specificity of the two Nbs determined by ELISA.

SDS-PAGE analysis confirmed that the recombinant proteins Mut-Nb1 and wild-type Nb were induced and expressed in *E. coli* BL21 (Figure 4A). The purified Mut-Nb1 and wild-type Nb proteins were detected in lanes 1 and 2, respectively, with apparent molecular weights of approximately 15 kDa, which were consistent with the theoretical molecular weights.

### 3.6. Affinity and Specificity Assessment

Binding affinities of Mut-Nb1 and wild-type Nb to HS and CAP were determined simultaneously in vitro via indirect ELISA, and nonlinear regression fitting was performed based on the one-site specific binding model (Figure 4B,C). Compared with the OVA negative control group, the OD_450_ values corresponding to HS exhibited a typical sigmoidal dose-dependent increase as the concentration of Mut-Nb1 gradually increased. The *K_D_* value of Mut-Nb1 against HS was calculated to be 65.87 nM, indicative of strong binding affinity between Mut-Nb1 and HS (Figure 4B). In contrast, Mut-Nb1 exhibited weaker binding to CAP, with a *K_D_* value of 608.90 nM. For wild-type Nb, the *K_D_* values of CAP and HS were 77.08 nM and 446.92 nM, respectively (Figure 4C). Comparative analysis revealed that Mut-Nb1 exhibited a 6.78-fold higher binding affinity for target HS but a 5.80-fold lower affinity for CAP relative to wild-type Nb.

Using the same indirect ELISA system, the binding specificity of the two Nbs toward HS, CAP, and HS structural analogs (CS and KS) was further evaluated (Figure 4D,E). The P/N values of Mut-Nb1 for CAP, CS, and KS were 1.87, 1.15, and 0.82, all below the threshold value of 2.1 for specific binding, whereas the P/N value for target HS reached 3.84 (Figure 4D). In comparison, the P/N values of wild-type Nb for CAP, CS, KS, and HS were 1.52, 4.31, 1.26, and 1.15, respectively (Figure 4E). Collectively, these results indicate that the specificity of Mut-Nb1 for recognizing the target HS was markedly improved by mutagenesis.

Furthermore, the cross-reactivity (CR%) was adopted to verify the specificity of the two Nbs (Table 5). Mut-Nb1 showed a CR of 10.87% toward CAP and less than 6.60% toward the HS structural analogs CS and KS, suggesting low non-specific cross-reactivity. Wild-type Nb showed a CR of 17.25% toward HS and < 10.14% toward all tested HS analogs. Taken together, these data demonstrate that Mut-Nb1 possesses high specificity for HS recognition.

## 4. Discussion

The recognition and binding of viruses to target cells constitute the initial step in viral infection, a process that requires interactions with cell surface attachment receptors. Numerous studies have demonstrated that HS, acting as an initial adhesion receptor, is involved in the attachment and entry of diverse pathogens, especially viruses, including DENV-2 [3], PCV2 [7], PRRSV [27], porcine deltacoronavirus (PDCoV) [28], vaccinia virus (VACV) [29], rabies virus (RV) [30], pteropine orthoreovirus (PRV) [31], and human adenovirus (HAdV) [32]. Accordingly, HS significantly influences viral pathogenicity, tissue tropism, and host range.

In this study, PDB: 7TJC was selected as the wild-type Nb scaffold [33] for two main reasons. Structurally, this native chloramphenicol-binding Nb features a typical ligand-binding mode that clamps the antigen between the CDR3 region and FR2. Meanwhile, the molecular size and intrinsic physicochemical characteristics of its original ligand, chloramphenicol, show moderate similarity to HS, endowing 7TJC with a naturally favorable pocket microenvironment for subsequent targeted sequence modification. In addition, its molecular docking score with HS was −7.30 kcal/mol (<−6.0 kcal/mol), preliminarily verifying that this Nb has potential binding capacity toward HS and is suitable as an ideal starting scaffold for affinity maturation and specificity reorientation. Currently, computer-aided techniques are widely applied in the rational design and molecular engineering of Nbs. Common computational tools include homology modeling, molecular docking, MD simulation, virtual mutation, and binding energy analysis, represented by classic software such as SWISS-MODEL, AutoDock, and GROMACS [34,35]. Moreover, machine learning has increasingly been applied to Nb sequence optimization [35]. Despite the capability of current computational methods to improve engineering efficiency and lower experimental costs, they present inherent limitations. First, these approaches rely heavily on high-resolution protein structures and reliable homologous templates, resulting in considerably lowered prediction accuracy for targets with complex modifications and high conformational flexibility. Second, conventional docking is typically static and fails to fully reflect the impacts of solvent environment and dynamic conformational transitions on polysaccharide–Nb recognition. Third, traditional virtual mutation strategies mainly concentrate on individual residue functions, neglecting inter-residue synergistic effects and underlying mechanisms of interface remodeling [36]. In contrast, the computational strategy proposed herein integrates molecular docking, MD simulation, binding energy decomposition, and multi-site cooperative mutation screening. This strategy enables systematic characterization of the binding interface between Mut-Nb1 and HS, allowing the identification of key residues and further optimization of intermolecular interactions. Notably, this approach is independent of any specific Nb framework. Therefore, the established workflow exhibits broad generalizability and can be readily extended to the rational molecular engineering of other target-specific Nbs.

The structure of Nbs comprises three CDRs and four FRs. In antigen recognition, CDR3 contributes the most significantly, followed by CDR2, FRs, and CDR1 [37,38]. The mutation design employed in this study follows this structure–function relationship. We found that the Phe47 residue in CDR2 formed only weak hydrophobic interactions with negatively charged HS via its hydrophobic side chain. Mutating this residue to a positively charged Arg significantly enhanced the electrostatic interaction between HS and Mut-Nb1. Although the Asp99 residue in CDR3 is negatively charged, its electrostatic contribution was only −0.33 kcal/mol in the alanine scanning analysis. Moreover, based on the results of the three-point combinatorial mutation screening, mutating Asp99 to Tyr was the most favorable for the binding between HS and Mut-Nb1, which was attributed to the ability of Tyr to provide both a new hydrogen bond donor and a hydrophobic aromatic ring. The Tyr108 residue is situated in a flexible loop region of CDR3. Importantly, the high conformational flexibility of loop regions often impairs the rigidity and complementarity of the binding interface, which is unfavorable for the formation of an optimal binding mode. After mutating Tyr108 to Pro, the unique rigid cyclic structure of Pro effectively restricts the conformational flexibility of this loop region, thereby stabilizing it within a conformational range favorable for molecular interactions.

In this study, static molecular docking analysis was first performed to characterize the initial recognition pattern between HS and Mut-Nb1. The docking score of −8.10 kcal/mol indicated the spontaneous binding propensity of the two molecules at the static structural level. The binding interface was collectively stabilized by polar interactions (hydrogen bonds and electrostatic interactions) mediated by Arg47 and Ser106, as well as a nonpolar π–sulfur interaction contributed by Phe37. Notably, distinct from the static docking scoring, subsequent MD simulations and MMPBSA decomposition calculations yielded a reliable overall binding free energy of −83.26 ± 3.06 kcal/mol. The numerical difference between the docking score and MMPBSA binding energy originates from their inherent calculation principles: docking relies on a single static conformation with simplified solvent conditions for rapid scoring, whereas MMPBSA averages thermodynamic parameters over dynamic trajectory frames under explicit aqueous environments, thus better reflecting the real binding state in solution. This MD-derived result verifies the favorable thermodynamic stability of the HS-Mut-Nb1 complex in aqueous solution. Furthermore, per-residue energy decomposition analysis confirmed that Arg47 serves as the dominant residue contributing to binding energy, while Ser106 and Phe37 play auxiliary roles in consolidating the binding interface. Collectively, these findings demonstrate that Arg47-mediated polar interactions act as the core driving force for complex stability throughout the entire binding process, spanning from the initial static docking pose to the final dynamic equilibrium state. Further experimental measurement of the *K*_D_ of the Arg47 mutant under different salt concentrations will help validate the critical electrostatic contribution of Arg47 and additionally verify the reliability of our MD simulation results. Experimental characterization confirmed that Mut-Nb1 binds to HS with a *K_D_* of 65.87 nM, representing a 6.78-fold higher affinity than that of wild-type Nb, along with a P/N value of 3.84 and a CR of <6.60%, collectively demonstrating its high affinity and specificity. Future cell-based binding assays using HS-positive and HS-negative cell lines could be performed to further validate the specific cellular targeting of Mut-Nb1. Additionally, competition assays with excess free HS can help evaluate nonspecific binding. These efforts will provide valuable insights into the cellular targeting mechanism of Mut-Nb1 and support its future applied research.

Research on Nbs in human virology has been extensive, exemplified by studies targeting the receptors of MERS-CoV [39] and SARS-CoV-2 [40]. In veterinary medicine, Nb research has also emerged as a promising trend. Cui et al. [41] utilized hybridoma technology to generate and characterize AN98, an antibody antagonist targeting the porcine growth hormone receptor (pGHR). Subsequent functional assays demonstrated that AN98 potently inhibited growth hormone-mediated signaling in both CHO-pGHR cells and porcine hepatocytes. Moreover, AN98 significantly reduced the secretion of insulin-like growth factor-1 (IGF-1) induced by growth hormone in porcine hepatocytes. Recently, Yang et al. [42] developed a bispecific Nb (BiNb-CD205) targeting the C-type lectin receptor (CD205), aiming to enhance antigen delivery and immune activation against foot-and-mouth disease virus (FMDV). In vitro experiments confirmed that BiNb-CD205 efficiently bound to porcine dendritic cells and localized to lysosomes, thereby promoting antigen uptake and processing. In vivo studies demonstrated that BiNb-CD205 elevated antibody titers, enhanced CD4^+^ and CD8^+^ T-cell responses, and promoted a balanced Th1/Th2 immune response. Furthermore, in a study on the zoonotic Nipah virus (NiV), Odchimar et al. [43] used computer-aided design to identify and obtain a high-affinity Nb targeting its fusion protein (NiVF). This work highlights the efficiency and precision of computer-aided design in guiding the rational design of Nbs.

By virtue of its high affinity and specificity, Mut-Nb1 holds promise as a molecular tool for studying HS function. First, its small size and single-domain structure render it suitable for super-resolution imaging and dynamic live-cell tracking, enabling the elucidation of the nanoscale distribution and dynamic changes of HS on the cell surface. Second, Mut-Nb1 can act as a viral adsorption inhibitor to specifically block the interaction between HS and viral particles. Furthermore, immobilization of this Nb allows the construction of an efficient affinity purification platform, which can systematically capture and identify the HS-interacting proteome, thereby facilitating the discovery of novel interacting factors and signaling regulatory nodes. The present study focused primarily on the rational mutation design and in vitro characterization of Mut-Nb1, laying the groundwork for subsequent investigations. Accordingly, exploring the aforementioned application potential will be a central focus of our future research.

## 5. Conclusions

In summary, this study developed the first mutant Nb targeting HS (HS-Mut-Nb1) through rational design and completed its systematic experimental characterization. This work not only provides a novel candidate molecular probe for HS function research but also offers a potential inhibitor for non-enveloped virus infections. Furthermore, the “computational simulation–experimental validation” strategy employed in this study provides a feasible reference for the rapid development of engineered Nbs.

## Figures and Tables

**Table 1:** Virtual alanine scanning of amino acid residues involved in the interaction between HS and wild-type Nb.

Mutation ^1^	Mutation Energy	Effect of Mutation	VDW ^2^ Term	Electrostatic Term	Entropy Term
Phe47 → Ala	1.69	Destabilizing	3.42	−0.04	0
Tyr59 → Ala	1.66	Destabilizing	3.66	−0.11	−0.15
Arg109 → Ala	0.93	Destabilizing	1.67	0.2	−0.01
Phe37 → Ala	0.91	Destabilizing	1.69	−0.04	0.11
Tyr108 → Ala	0.56	Destabilizing	1.83	−0.03	−0.43
Pro111 → Ala	0.44	Neutral	0.93	0.02	−0.05
Asp99 → Ala	0.34	Neutral	0.95	−0.33	0.04
Ile57 → Ala	0.23	Neutral	0.36	0.01	0.05
Gly35 → Ala	0.04	Neutral	0.11	0.01	−0.02
Ser100 → Ala	0.01	Neutral	0.01	0	0

^1^ The top five and bottom five residues ranked by mutation energy in descending order (total 10 residues) were selected. ^2^ VDW: van der Waals.

**Table 2:** Single-site saturation mutagenesis of the six key amino acid residues.

Mutation ^1^	Mutation Energy	Effect of Mutation	VDW ^2^ Term	Electrostatic Term	Entropy Term
Asp99 → Phe	−0.88	Stabilizing	−1.27	−0.48	0
Asp99 → Gln	−0.62	Stabilizing	−1.04	−0.25	0.03
Asp99 → Asn	−0.26	Neutral	−0.13	−0.38	0
Asp99 → Tyr	−0.25	Neutral	−0.07	−0.42	−0.01
Asp99 → Leu	−0.18	Neutral	0.02	−0.36	−0.01
Tyr108 → Phe	−0.11	Neutral	0.02	−0.01	−0.15
Phe47 → Arg	−0.02	Neutral	−1.05	−0.23	0.78
Tyr108 → Pro	−0.01	Neutral	0.74	−0.07	−0.43
Asp99 → Cys	0.30	Neutral	0.93	−0.35	0.01
Tyr108 → Val	0.40	Neutral	1.06	−0.01	−0.16

^1^ The top 10 mutant variants are listed in ascending order of mutation energy. ^2^ VDW: van der Waals.

**Table 3:** Double-site saturation mutagenesis of the six key amino acid residues.

Mutation ^1^	Mutation Energy	Effect of Mutation	VDW ^2^ Term	Electrostatic Term	Entropy Term
Asp99 → Tyr Tyr108 → Arg	−2.02	Stabilizing	−3.12	−0.6	−0.2
Asp99 → Phe Tyr108 → Arg	−1.92	Stabilizing	−2.91	−0.65	−0.18
Phe47 → Arg Asp99 → Arg	−1.44	Stabilizing	−3.59	−0.58	0.8
Asp99 → Asn Tyr108 → Arg	−1.39	Stabilizing	−1.96	−0.55	−0.17
Phe47 → Arg Asp99 → Tyr	−1.06	Stabilizing	−2.83	−0.55	0.79
Asp99 → Tyr Tyr108 → Pro	−0.91	Stabilizing	−0.57	−0.54	−0.44
Phe47 → Arg Asp99 → Phe	−0.78	Stabilizing	−2.13	−0.66	0.77
Asp99 → HIS Tyr108 → Arg	−0.64	Stabilizing	−0.48	−0.55	−0.15
Asp99 → Cys Tyr108 → Arg	−0.60	Stabilizing	−0.37	−0.57	−0.16
Phe47 → Arg Asp99 → Met	−0.55	Stabilizing	−1.84	−0.54	0.8

^1^ The top 10 mutant variants are listed in ascending order of mutation energy. ^2^ VDW: van der Waals.

**Table 4:** Three-site combinatorial mutations derived from single-site saturation mutations.

Mutation ^1^	Mutation Energy	Effect of Mutation	VDW ^2^ Term	Electrostatic Term	Entropy Term
Phe47 → Arg Asp99 → Tyr Tyr108 → Pro	−1.61	Stabilizing	−2.93	−0.76	0.3
Phe47 → Arg Asp99 → Phe Tyr108 → Phe	−1.55	Stabilizing	−3.28	−0.71	0.56
Phe47 → Arg Asp99 → Phe Tyr108 → Pro	−1.49	Stabilizing	−2.70	−0.76	0.3

^1^ The top three combinatorial mutants are listed in ascending order of mutation energy. ^2^ VDW: van der Waals.

**Table 5:** Identification of the cross-reactivity of Mut-Nb1 and wild-type Nb.

Antigen	Mut-Nb1		Wild-Type Nb	
	EC_50_ (nM)	CR (%)	CR (%)	CR (%)
HS	65.87	100	446.92	17.25
CAP	608.90	10.87	77.08	100
CS	>1 × 10^3^	<6.60	<7.6 × 10^2^	<10.14
KS	>1 × 10^3^	<6.60	<7.6 × 10^2^	<10.14

HS: Heparan Sulfate; CAP: chloramphenicol; CS: chondroitin sulfate; KS: keratan sulfate; EC_50_: half-maximal effective concentration; CR (%): cross-reactivity.

## Data Availability

The original contributions presented in this study are included in the article. Further inquiries can be directed to the corresponding authors.
